# P-1929. Changing Characteristics of Cryptococcal Infections and Outcomes: A Single-Center Retrospective Cohort

**DOI:** 10.1093/ofid/ofaf695.2098

**Published:** 2026-01-11

**Authors:** Charis C Hodges, Kenneth D Long, Gerald McGwin, Todd P McCarty, Peter G Pappas

**Affiliations:** The University of Alabama at Birmingham, Huntsville, AL; The University of Alabama at Birmingham, Huntsville, AL; University of Alabama at Birmingham, Birmingham, Alabama; University of Alabama at Birmingham, Birmingham, Alabama; University of Alabama at Birmingham, Birmingham, Alabama

## Abstract

**Background:**

Cryptococcal meningitis and pneumonia have historically been AIDS-defining illnesses but are increasingly seen in non-HIV pts with and without immunocompromise. This single-center retrospective cohort study aims to describe differences in demographics, comorbidities, infection sites, treatment, and outcomes of pts infected with *Cryptococcus spp.* since 1996.

**Table 1.**
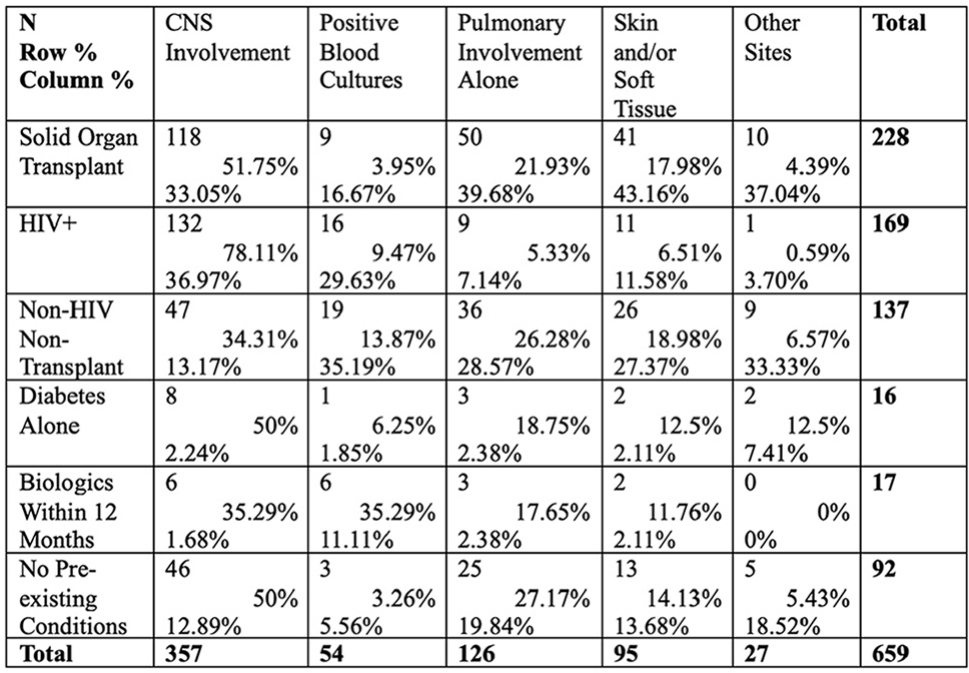
Site of infection and comorbidity frequencies.

**Table 2.**
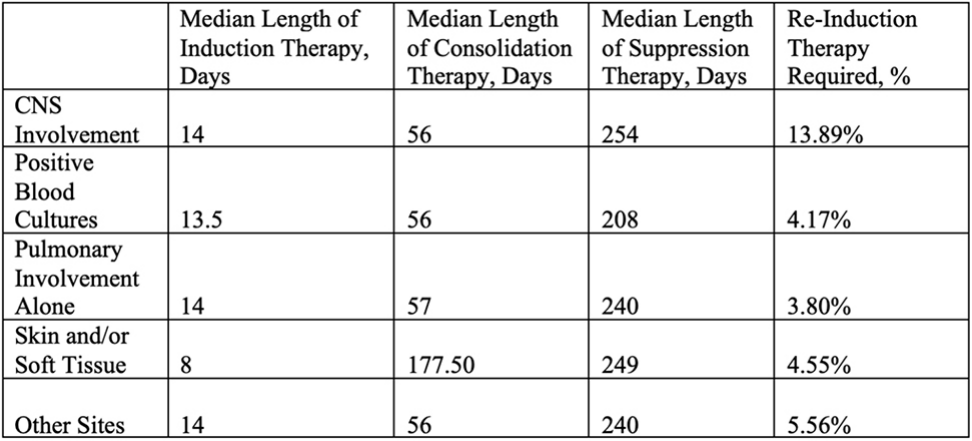
Length of therapy by site of infection.

**Methods:**

659 pts were identified from 1996-2023 using positive *Cryptococcus* serum and CSF antigen titers as well as tissue, blood, and CSF cultures. Pediatric pts were excluded. Chart review was performed to determine comorbidities, infection sites, treatment, and outcomes. Pts were divided by solid organ transplant recipients (SOTs), HIV+, diabetes (DM) alone, those who received biologics within the past 12 mos, other non-HIV/non-transplant, and no pre-existing conditions.

**Figure 1.**
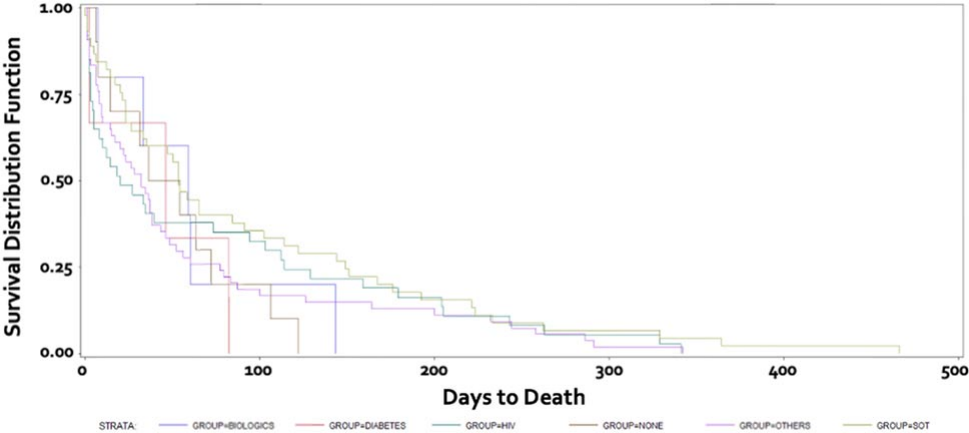
Kaplan-Meier curve by comorbidities.

**Figure 2.**
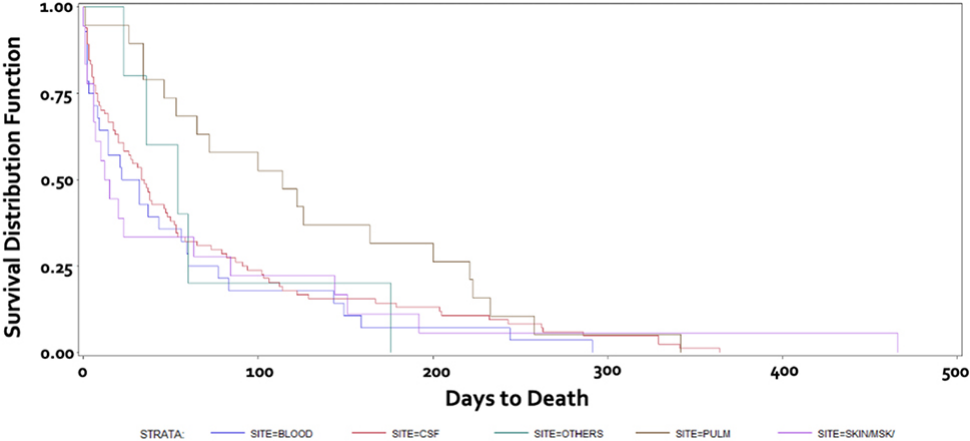
Kaplan-Meier curve by site of infection.

**Results:**

See Tables 1 & 2 and Figures 1 & 2.

Mean days from symptom onset to diagnosis was 30 for SOTs; 25 for HIV+ pts; 49 for non-HIV non-transplant pts; 60 for pts with DM alone; 42 for pts who received biologics within the past 12 mos; and 96 for those with no pre-existing conditions (p< 0.0001).

Mean days of follow-up was 752 for SOTs; 879 for HIV+; 452 for non-HIV non-transplant pts; 605 for DM alone; 634 for those who received biologics in the past 12 months; and 888 for those with no pre-existing conditions (p=0.0066).

Among the different groups analyzed, all had a male predominance; the percentage of females varied from 43.75% in those who received biologics to just 19.76% in those with HIV. All groups had predominantly white pts except for HIV+, which was predominantly African American.

1-year mortality was highest in the non-HIV non-transplant group at 55.10%, with the lowest 1-year mortality in those with no pre-existing conditions at 15.38% (p< 0.0001). SOT pts had 23.04% mortality, DM alone had 27.27%, HIV+ had 29.84%, and those who received biologics had 31.25%.

**Conclusion:**

This study highlights important variations among demographics, sites of infection, treatment, and outcomes among *Cryptococcus* spp. infections among different subgroups of pts. Most importantly, differences in time to diagnosis and one-year mortality are identified. Further research is needed to explore these differences, identify infections earlier, and improve outcomes.

**Disclosures:**

Todd P. McCarty, MD, Basilea: Grant/Research Support|Cidara: Grant/Research Support|F2G: Grant/Research Support|Mundipharma: Grant/Research Support|Pfizer: Advisor/Consultant|Scynexis: Grant/Research Support Peter G. Pappas, MD, Astellas: Grant/Research Support|Basilea: Advisor/Consultant|Basilea: Grant/Research Support|F2G: Advisor/Consultant|Gilead: Grant/Research Support|Melinta: Advisor/Consultant

